# Research Progress Based on Regulation of Tumor Microenvironment Redox and Drug-Loaded Metal-Organic Frameworks

**DOI:** 10.1155/2022/7302883

**Published:** 2022-07-22

**Authors:** Tong Xu

**Affiliations:** State Key Laboratory of Inorganic Synthesis and Preparative Chemistry, College of Chemistry, Jilin University, Changchun 130012, China

## Abstract

The process of tumor growth and deterioration is accompanied by increased oxygen free radicals, high glutathione concentration, hypoxia, and poor drug targeting during treatment, limiting the treatment of tumors. Metal-organic framework (MOF) preparations are continuously being developed and applied in tumor therapy. In this paper, the design and application of reactive oxygen species (ROS) and redox drug-loaded MOF preparations are reviewed. Moreover, the research challenges and application prospects of MOFs in tumor therapy are also discussed.

## 1. Introduction

The tumor is a symptom of a severe disease that threatens human health. There is a significant difference between the physiological characteristics of tumor tissues and normal tissues. The commonly used treatments for tumors include chemotherapy, radiotherapy, photothermal therapy, and surgical resection. However, there are various challenges associated with the current clinical tumor treatment, which is consistently ineffective and has various side effects. For example, tumor metastasis makes complete tumor removal through conventional surgical resection difficult [1]. As the primary tumor treatment method, chemotherapy is mainly used to control tumors by applying drugs that interfere with cell proliferation and remove tumor cells [2]. However, in addition to killing tumor cells, traditional chemotherapy has strong side effects against the normal cells, tissues, and organs of the human body. Therefore, with the use of chemotherapy, tumor cells become multidrug resistant, which significantly reduces chemotherapy efficacy [3].

For a long time, researchers have been committed to identify and develop new functional materials and advanced technical methods to deliver drugs to tumor tissues safely and effectively and to achieve the best therapeutic effects [4, 5]. The tumor microenvironment (TME) is a complex local tissue environment where tumor cells are located. The TME not only plays a vital role in the occurrence, growth, and metastasis of tumors but also mediates tumor resistance through various mechanisms [6]. The TME in tumor tissues is significantly different from the microenvironment of healthy tissues for the following reasons. First, there are high levels of reactive oxygen species (ROS) in tumor cells, such as hydrogen peroxide (H_2_O_2_), hydroxyl radicals (•OH), and singlet oxygen (^1^O_2_) [7]. Second, there are high concentrations of reducing substances in tumor tissues. This study [8] reported that in tumor tissues, the level of GSH is at least 4 times higher than that in ordinary tissues, and the intracellular GSH content (2-10 mmol/L) is almost 100-1000 times higher than that in the extracellular area (2-10 *μ*mol/L), resulting in the formation of redox gradients across cells. Thirdly, tumor tissues have a weak acidic environment, with a pH value between 6.2 and 6.9 [9]. Finally, the ATP content in tumor cells is greater than that in ordinary cells [10], and the oxygen concentration is much lower than that of ordinary cells [11]. Based on these distinctive physiological features of the tumor tissue described above, the introduction of drug carriers to TMEs is anticipated to effectively address these issues by providing stable *in vivo* transport, targeted delivery, high permeability, and targeted drug release by the medicine carriers.

MOFs are a novel type of crystalline and ordered porous substances formed by connecting metal ions and organic ligands with coordination bonds. They have great potential in biological applications due to their tunable dimension, shape, softness of porous crystals, and inherent biodegradability [12–14]. Furthermore, as a structurally diverse and rapidly developing vehicle, MOFs have been widely recognized as forward-looking tools to overcome the challenges of drug delivery [15–19]. MOFs have made significant progress in inducing ROS, reducing GSHs and increasing drug loadings. This review summarizes the development and research progress in this field that has been reported in recent years.

## 2. ROS-Responsive MOFs and MOF-Derivative Drug Carrier

ROS is an array of highly active oxygen-containing derivatives, such as superoxide anion (O^2−^), HO•, H_2_O_2_, and ^1^O_2_ [20]. As the main molecules produced when the body is under oxidative stress, they play important roles in a variety of physiological and pathological processes, mainly in the mitochondria, peroxisomes, and endoplasmic reticulum in cells, and they are also involved in various extracellular biochemical reactions. Tumor cells have higher levels of ROS than normal cells, with crucial roles in tumorigenesis. However, paradoxically, there is increased expression of antioxidant proteins in cancer tissues, inhibiting ROS production [21–23]. Furthermore, increasing evidence has confirmed that ROS induction can cause apoptosis by disrupting the metabolism of cancer cells. Recently, MOFs have been used as a unique platform to integrate photosensitizers. Compared with traditional materials, MOF materials have the designable structures, large specific surface areas, and permanent porosities that allow them to effectively prevent light bleaching, polymerization triggering, self-quenching, and water solubility. Meanwhile, the production of ROS and PDT efficiency can be greatly enhanced. In addition, the inherent sensitivity and responsiveness of the MOF skeleton allows it to preferentially accumulate in tumor regions, increasing the therapeutic concentration of loaded drugs in tumor cells. Based on the difference in ROS content inside and outside the target cells, ROS-responsive MOF drug carriers constructed from ROS-sensitive materials have been extensively studied for the control of the intracellular release of drug molecules (Table 1).

**Figure 1:**
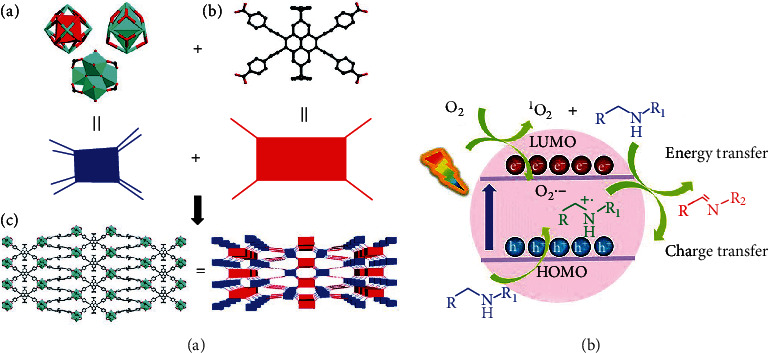
(a) The construction of PCN-822 (M) (M = Zr, Hf). (b) Proposed mechanisms of oxidation of amines [27].

**Figure 2:**
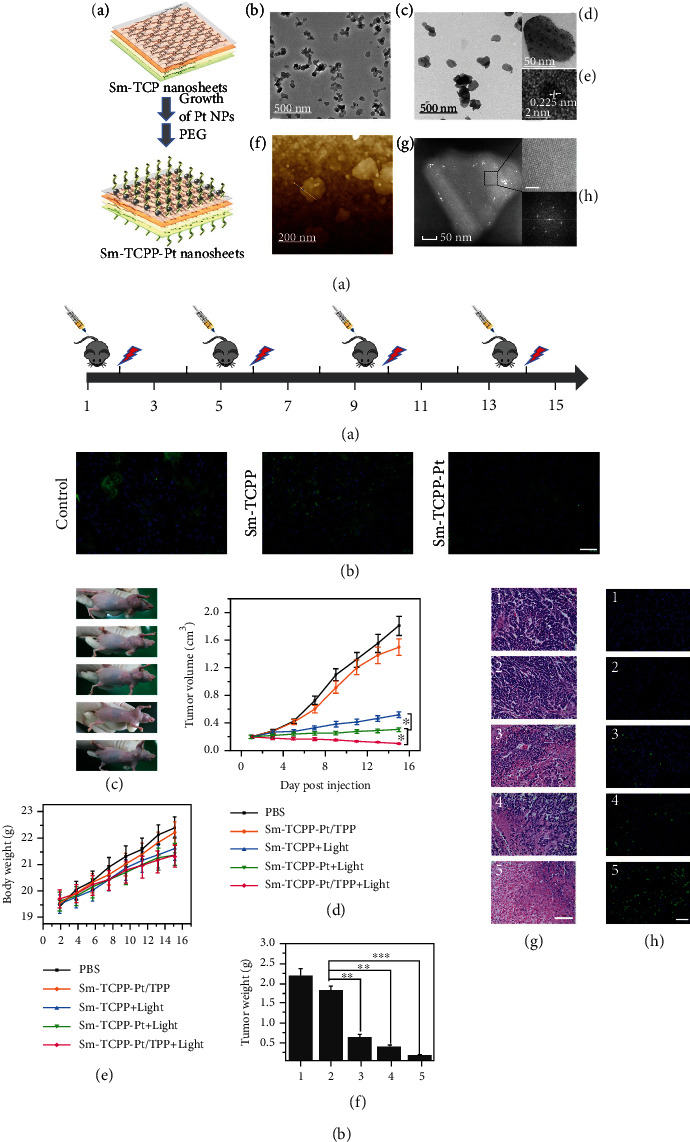
(a) Preparation of Sm-TCPP-Pt nanosheets and its TEM images, high-resolution TEM images, AFM image, and scanning transmission electron microscope (STEM) image. (b) Anticancer effect assessment of nanoagents in vivo [28]. *Copyright © 2020, American Chemical Society.*

Sulfonamides (SAs) are prone to be deposited in cancer cells. At the same time, bovine serum albumin (BSA) has a long half-life circulation and tissue compatibility; thus, it could be an ideal drug carrier. Consequently, BSA- and SA-embellished iron porphyrin MOFs (BSA/SA–NMOF), constructed by Zhu et al. [24], showed good biocompatibility and a long circulation time, which are similar properties to those of the drugs used for targeting cancer cells due to the existence of both SA and BSA. *In vitro* experiments showed that BSA/SA–NMOF could induce 4T1 cells to produce a large amount of ROS, leading to cell death, and *in vivo* experiments also confirmed that BSA/SA–NMOF had a good tumor-inhibiting effect [20]. Additionally, Y. Wang et al. [25] loaded photosensitizers (PSs) into MOF-199, a Cu(II) carboxylic acid-based MOF. Following the ingestion of MOF-199 loaded with photosensitizer by cells, Cu(II) in MOF effectively eliminated the endogenous GSH. Moreover, it induced the disintegration of MOF-199 to deliver coated PSs and promote ROS production in cancer cells. *In vitro* and *in vivo* tests confirmed the high-efficiency antitumor cell effect of the synthesized and modified MOF-199. Wang et al. [26] prepared ultrafine porphyrin MOF nanodots (MOF-QDs) for photodynamic therapy (PDT) of tumors using a simple method. The pharmacokinetic results showed that ultra-MOF-QDs could effectively accumulate at the tumor site and then quickly be eliminated by the kidney. Compared with the precursor NMOFs, MOF-QDs exhibited a better PDT effect by generating the ROS levels at two times higher and at one-third of the NMOF IC50 values, suggesting better antitumor potential. Zhang et al. [27] synthesized two porous MOFs, PCN-822 (M) (M = Zr, Hf), consisting of 4, 5, 9, 10-(region K)-substituted pyrene ligands, viz., 4, 4′, 4^″^, 4^‴^-(2, 7-di-tert-butylpyrene-4, 5, 9, 10-tetraaryl) tetra (ethyl-2, 1-diaryl) tetrabenzoate (BPETB4) (Figure 1). The compound showed a good response reaction to visible light within the range of 225 to 650 nm to efficiently generate ROS. Gao et al. [28] designed and synthesized a novel two-dimensional MOF (Sm-TCPP) by packing together transition metal ion (Sm^3+^) and PS (TCPP) nanosheets (Figure 2). The nanosheets were approximately 100 nm in diameter and less than 10 nm in thickness and loaded with platinum nanoenzymes mimicking catalase (CAT). ^1^O_2_ generation is significantly enhanced by improving its physical and chemical features and enhancing the cross reactions between systems. More interestingly, the Pt nanoenzyme mimicking CAT over the Sm-TCPP nanosheet effectively changed H_2_O_2_, overexpressed in the cancer microenvironment, into O_2_, thus alleviated cancer hypoxia. In addition, triphenylphosphine (TPP) introduction into the Sm-TCPP-Pt system could develop a PDT system targeting the mitochondria and self-providing oxygen. The experimental results of the *in vivo* and *in vitro* models of MCF-7 breast cancer demonstrated that Sm-TCPP-Pt/TPP can alleviate cancer hypoxia, and the ROS generated near the mitochondria of cells could induce apoptosis.

**Figure 3:**
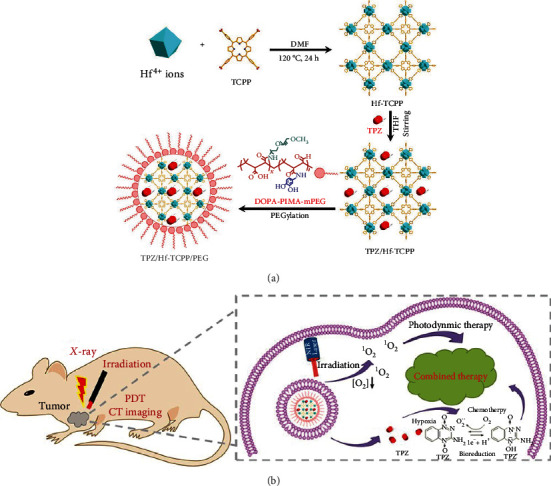
(a) The synthesis of TPZ/Hf/TCPP/PEG. (b) In vivo synergetic photodynamic and hypoxia-activated therapy of TPZ/Hf/TCPP/PEG [31]. *Copyright © 2022, American Chemical Society.*

^1^O_2_ is a well-known ROS in PDT and other fields [29]. W. Zhang et al. [30] synthesized a novel singlet oxygen-producing system named bio-MOF-1&RCs by exchanging the cationic ruthenium complexes (RCs) into the anionic bio-MOF-1 with a good ^1^O_2_ producing function. M. Liu et al. [31] prepared a multifunctional Hf-porphyrin NMOF nanocarrier named Hf-TCPP with a large loading amount, good crystallinity, and a large BET surface area. Moreover, a hypoxia-activated prodrug (tirapazamine, TPZ) was loaded into Hf-TCPP. To enhance the dispersibility, blood circulation time, and structural stability of the medicine carrier in the biological medium and to control the release of the drug, TPZ/Hf-TCPP/PEG was developed by introducing dopamine-derived polyethylene glycol (DOPA-PIMA-mPEG) by chemical modification. TPZ/Hf-TCPP/PEG generated ^1^O_2_ and induced apoptosis during light exposure (Figure 3). Lan et al. [32] synthesized a new type of nMOF-Ti-TBP with a chain of Ti-oxo secondary building units (SBUs) and photosensitive 15, 10, 15, 20-(p-benzoate) porphyrin ligand (TBP). Light irradiation triggered Ti-TBP to generate ^1^O_2_ and transferred electrons from stimulated TBP∗ species to Ti^4+^-based SBUs to supply TBP^•+^ ligands and Ti^3+^ centers, thereby promoting the generation of H_2_O_2_ and •OH. *In vivo* experiments confirmed that Ti-TBP had a significant effect on solid tumors in mice, with a 98% cancer regression rate and a 60% cure rate. ZrMOF NC is an effective microwave-sensitive agent. Fu et al. [33] prepared novel and ductile Mn-doped zirconium MOF (Mn-ZrMOF) nanocubes with an average particle dimension of approximately 60 nm using a one-pot hydrothermal process. More importantly, these Mn-ZrMOF NCs generate substantial ROS and hydroxyl radicals under microwave irradiation, effectively restraining the growth of tumor cells *in vitro* and *in vivo*.

## 3. Reduction-Responsive Nanodrug Carriers

Tumor tissue has a strong reducing environment within the cells due to its abnormal metabolism. In cancer cells, glutathione (GSH, 2-10 mmol/L) intracellular levels were present at 1000 times higher than extracellular levels (2-20 *μ*mol/L) and 4 times higher than normal intracellular concentrations. Moreover, GSH was even more than 10 times higher in some medicine-resistant cancer cells [34]. Therefore, the difference in the reducibility inside and outside of tumor cells provides a platform for designing reduction-responsive nanodrug carriers to release drugs inside of tumor cells. The common approach is to use nanomaterials to decrease GSH levels in cells. For example, Cramer et al. used cysteines to decrease L-cysteine, a crucial part of GSH production, to indirectly reduce intracellular GSH levels [35]. Lin et al. and Fan et al. [36, 37] also revealed that manganese dioxide nanosystems improved cancer treatment by decreasing intracellular GSH levels. In addition, Tristao et al. utilized Cu(II)-graphite carbon nitride to regulate ROS [38]. In recent years, more and more promising studies have been conducted in the area of medicine delivery which utilizes MOF drug carriers. Cu, Fe, and Mn, as the commonly used metal ions in the structure of MOF drug carriers, have been extensively reported (Table 2).

**Figure 4:**
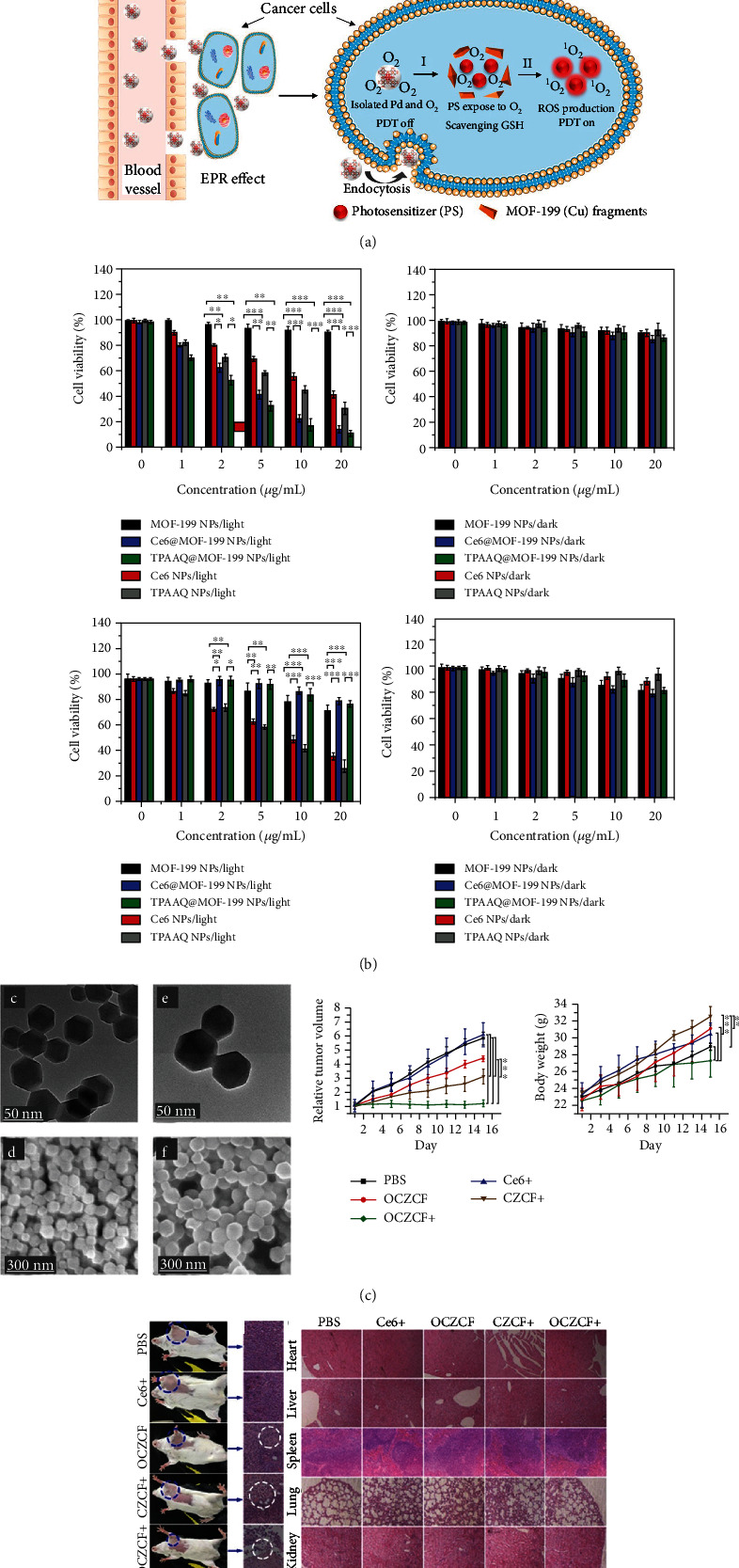
(a) The synthesis of PS@MOF-199 and F127-coated PS@MOF-199 (PS@MOF-199 NPs) and quench and trigger of photosensitization originated from PS@MOF-199 NPs in the tumor microenvironment. (b) Viability of HepG2 (up) and NIH-3T3 cells (bottom) upon incubation with Ce6 NPs, Ce6@MOF-199 NPs, TPAAQ NPs, and TPAAQ@MOF-199 NPs under white light or dark [25]. *Copyright © 2022, American Chemical Society.* (c) TEM images and SEM images of Cu/ZIF-8 and OCZCF. (d) 4T1 tumor-bearing BALB/c mice were used for in vivo antitumor experiments [39]. *Copyright © 2019, American Chemical Society.*

Zhang et al. [34] designed and created a nano-MOF (Cu(II)-metalated nano-MOF (CuL-[AlOH]_2_)*_n_* (MOF-2, H_6_L = mesotetrakis (4-carboxylphenyl) porphyrin)) with Cu(II) as the active MOF kernel. Cu(II) could combine and uptake GSH specifically to directly reduce the cell GSH concentration and elevate ROS levels. Notably, this MOF caused apoptosis of mouse breast tumor cells with therapeutic effects comparable to camptothecin (CPT). Y. Wang et al. [25] synthesized PS@MOF-199 NPs with Cu(II) carboxylate-based MOF-199 as a carrier and evaluated the GSH scavenging function with HepG2 cells and 3T3 cells (Figures 4(a) and 4(b)). They found that Cu(II) MOF-199 could effectively scavenge intracellular glutathione after the endocytosis of PS@MOF-199 NPs by cells. Thus, the ROS produced by PS are not depleted by glutathione. Xie et al. [39] designed and synthesized a new biodegradable treatment system named O2-Cu/ZIF-8@Ce6/ZIF-8@F127 (OCZCF) to address the overexpression of GSH within the TME (Figures 4(c) and 4(d)). Experiments with L929 (mouse fibroblast cells) and 4T1 cells (mouse breast cancer cells) showed that OCZCF could achieve GSH depletion and increase the O^2-^ content, causing apoptosis. Meanwhile, *in vivo* assays confirmed that OCZCF significantly reduced solid tumor volume within 4T1 tumor-bearing BALB/c mice.

Wang et al. [40] prepared Janus nanocomposites (UPFB) for biocatalysts by incorporating core-shell-shell upconversion nanoparticles (UCNPs, NaYF4: 20% Yb,1% Tm@NaYF4:10% Yb@NaNdF4) and an iron-containing zirconium porphyrin metal-organic backbone [PCN-224 (Fe)]. Cellular assays confirmed that UPFB could deplete excess GSH inside the cells and promote ROS production. In addition, Fe^3+^ in UPFB behaves as a catalytic peroxidase-like nanoenzyme that catalyzes H_2_O_2_, O_2_ generation, TME hypoxia alleviation, and apoptosis. D. Zhu et al. [41] synthesized multifunctional nanoplatform- (TUDMP-) based NPs by sequentially integrating the photosensitizer TCPP, anticancer drug doxorubicin (DOX), MnO_2_, and hydrophilic polyethylene glycol (PEG) into the initial UIO-66 carrier based on a TUDMP with a multivariate porphyrin-nMOF core and MnO_2_ shell. *In vivo* experiments confirmed that TUDMP NPs could accumulate in large quantities at the tumor sites. The MnO_2_ shell layer effectively catalyzes the hydrolysis of H_2_O_2_ to generate O_2_ and depletes GSH, thereby contributing to cytotoxic ROS production through photosensitizers in laser irradiation. Importantly, the disintegration of the MnO_2_ shell layer could facilitate the liberation of loaded DOX and enable synergistic chemotherapy-PDT treatment. Induced disturbance of redox homeostasis often leads to cellular oxidative damage, inhibiting cancer cell proliferation and tumor regression. Therefore, silk fibroin-wrapped (capped) metal-organic backbone nanosystems (NS@TPZ (NST) NPs) with Fe(III) coordinated with 4, 4, 4, 4-(porphyrin-5, 10, 15, 20-tetra alkyl) tetra benzoic acid (TCPP) were synthesized by the one-pot method. Fe(III) in NST could effectively react with tumor GSH to generate Fe(II) and glutathione disulfide (GSSG). Experiments using 4T1 cells and 4T1 tumor-bearing mice revealed that NS@TPZ (NST) NPs effectively reduced the GSH content of tumor cells. Moreover, increased ROS levels induced abnormal mitochondrial membrane permeability that triggered tumor cell death [42].

**Figure 5:**
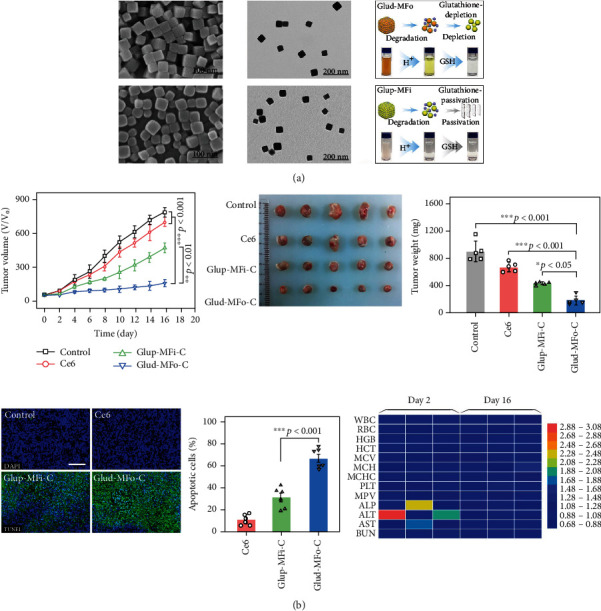
(a) Characterizations of Glup-MFi and Glud-MFo nanoparticles. (b) In vivo PDT assessments of control, Ce6, Glup-MFi-c, and Glud-MFo-c [44]. *Copyright © 2020, American Chemical Society.*

Multidrug resistance (MDR) is the primary reason for the ineffectiveness of chemotherapy. J. Xu et al. [43] developed a Cu^2+^-based organic framework (COF) for treating a multidrug-resistant tumor. They used it to deliver glucose oxidase (GOx) and adriamycin (DOX) (COF/GOx/Dox), in which GOx catalyzed H_2_O_2_ generation from glucose. At the same time, Cu^2+^ could react with glutathione and convert to Cu^+^, leading to the depletion of glutathione and intracellular accumulation of ROS. Later, Cu^+^ and H_2_O_2_ initiated the Fenton reaction to produce -OH, disrupting the redox balance in cancer cells. These factors promote cells and lead to a significant enhancement of anticancer properties *in vitro* and *in vivo*. D. Wang et al. [44] designed a multicomponent self-assembled PDT nanoformulation called Glud-MFo-c (Figure 5). The multicomponent coordination, *π*-*π* stacking, and electrostatic interactions among the metal ions, photosensitizers, and bridging linkers could generate homogeneous nanoparticles under the protection of biocompatible polymers. 4T1 and HeLa cell assays confirmed that Glud-MFo-c could effectively reduce the GSH content, amplify ROS production, and induce apoptosis. In addition, mouse experiments confirmed the growth inhibitory properties of Glud-MFo-c against tumors.

Ferroptosis and starvation therapy can also treat tumors. Glucose oxidase catalyzes the production of H_2_O_2_ from glucose, causing ferroptosis and glucose depletion in tumor cells, which is also called starvation therapy [45]. Tian et al. [46] synthesized a cancer cell membrane-covered cascade nanoreactor NMIL-100 using iron MOF and glucose oxidase for iron death-starvation anticancer therapy. When the nanoreactor reaches the tumor site, the GSH with high concentration reduces Fe^3+^ to Fe^2+^, and GOx catalyzes glucose oxidation to generate H_2_O_2_, consuming glucose in the cancer cells. Furthermore, Fe^2+^ and H_2_O_2_ generate highly toxic -OH radicals by the Fenton reaction, causing ferroptosis. HKUST-1 is a MOF with a molecular linkage of benzene-1,3,5-tricarboxylic acid dimer, and it is GSH-responsive with drug delivery functions. Thus, Tian et al. [47] designed and synthesized HKUST-1 cascade nanocatalysts (Sol/Mel@HKUST-1 NPs) loaded with Sol and a COX-2 inhibitor called ML. *In vitro and in vivo* experiments established that Sol/Mel@HKUST-1 NPs could decrease the GSH level, increase the ROS and LPO levels, reduce the expression of COX-2 and GPX4, activate PINK1/Parkin, and cause ferroptosis in tumor cells, all of which lead to significant therapeutic effects.

## 4. Research Progress of MOF Drug Loading

Due to the complexity of the cancer microenvironment and the inherent defects of the drug, which include poor responsiveness and easy drug resistance, its therapeutic effect has been seriously compromised [48]. Therefore, MOF drug loading has become a hotspot to improve tumor efficacy and reduce adverse drug reactions, due to its advantages such as easy modification and strong adsorption (Table 3).

Gao et al. [49] packaged pentafluorouracil into zeolitic imidazolate framework (ZIF), resulting in the loading efficiency increasing to 51%. Du et al. [50] loaded pentafluorouracil into MOF-In1, whose pores are negatively charged. As a result, zinc ions entered the pores when their intracellular concentration was high, corresponding to the release of the pentafluorouracil drug under the electrostatic effect. Finally, M. Wu et al. [51] used MOFs to encapsulate the photothermal conversion material of polypyrrole nanoparticles (PPy NPs) and the chemotherapeutic drug pentafluorouracil to achieve the combination of chemotherapy and photothermal effects.

Chen et al. [52] employed MOF nanoparticles to load the anticancer drug doxorubicin hydrochloride (DOX) and modified an ATP-responsive polyacrylamide/DNA hydrogel on the surface of MOFs. The crosslinking of this hydrogel is achieved by nucleic acid duplexes containing ATP-resistant aptamers inside a caged structure. The surface-modified hydrogel bridging unit of MOFs is decomposed due to the formation of an ATP-aptamer compound, which separates the hydrogel shell and releases DOX. F. Zhang et al. [53] covalently crosslinked DOX with amino functional groups to aldehyde functional groups on MOFs by forming Schiff bases. Thus, the high-efficiency loading of drug molecules on MOFs was achieved, and the drugs are released under acidic pH conditions. Using the in situ growth method, R. Chen et al. [54] used an MOF to wrap photosensitizer graphical carbon nitride (g-C3N4) nanosheets. Then, DOX was loaded by the pore wrapping method to obtain the combined photodynamic and chemotherapy treatment system. The framework benefits from the inclusion and controllability of the encapsulated material achieved by the MOF coprecipitation method and the large porosity of the MOF shell. In addition, MOFs can also be used to deliver other drugs, such as valsartan [62], curcumin [63], and camptothecin [55], thereby achieving a better therapeutic effect than small molecule drugs. Dong et al. [55] investigated the anticancer effects of a cannabidiol-loaded magnesium gallate MOF (CBD/Mg-GA). They showed that CBD/Mg-GA MOF slowly degraded under physiological conditions with the continuous release of GA and CBD. CBD induces mitochondrial dysfunction and ROS production, triggering an apoptotic cascade in glioma cells by regulating the expression of NF-*κ*B. Li et al. [56] found that the surface modification of nano-MOFs with lipids can preferably control the degradation of drugs in biological media. When loaded with anticancer medicines such as Gem-MP (gemcitabine-monophosphate), melamine iron nano-MOFs act as “Trojan horses,” which carry drugs into tumor cells to destroy them. Notably, nano-MOFs coated with polyethylene glycol (PEG) were captured by macrophages.

MOFs assembled with metalloporphyrin derivatives as ligands can be loaded with prodrug molecules such as nitrosothiols [57]. Under near-infrared illumination, the MOFs deliver photothermal therapy, while the loaded nitrosothiols can be heated to produce nitric oxide gas for the treatment of tumors. Yang et al. [64] designed a phase transfer method to construct PEGylated one-dimensional MOFs in a single step. This method is simple and easy to implement and can be applied to various metal ions. Such one-dimensional MOFs have excellent dispersibility and a long blood circulation time. Moreover, they can achieve charge reversal in slightly acidic tumor environments, which directly improves the retention and enrichment of medicine carriers at cancer sites. Instead of using high photothermal temperature to destroy tumors, gambogic acid (a heat shock protein inhibitor) can inhibit the expression of HSP90 (a protein related to thermotolerance), which efficiently induces apoptosis in cancer cells by low-temperature heating.

**Figure 6:**
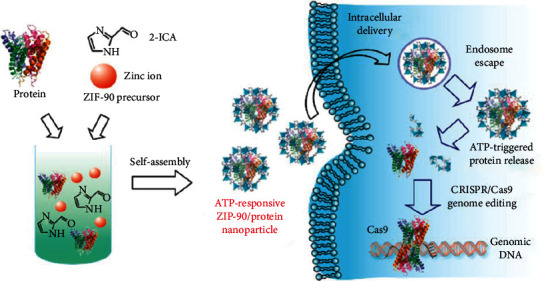
The self-assembly of ZIF-90/protein nanoparticle and ATP-triggered protein release from ZIF-90 nanoparticle inside cells [61]. *Copyright © 2019, American Chemical Society.*

Alsaiari et al. [58] used ZIF-8 to encapsulate Cas9 and efficiently deliver it into cells. ZIF-8 is degraded due to ligand protonation in the acidic environment of lysosomes. As a result, the lysosomal escape and release of Cas9 are enabled, and the function of Cas9 cleavage genes can be maintained. Another MOF named ZIF-90 can also encapsulate the Cas9 protein, presenting it to the tumor cytoplasmic matrix. The ATP molecule within the cytoplasm has a strong coordination effect with Zn^2+^ of ZIF-90, which induces ZIF-90 to degrade and release the Cas9 protein [61] (Figure 6). It also cuts tumor cell genes, thereby achieving the gene silencing effect. Shen et al. [59] wrapped DNA zyme with ZIF-8 to deliver it into cells and released DNA zyme and Zn^2+^ cofactor through lysosomal escape to achieve gene therapy. After the MOFs adsorb siRNA, they can transport siRNA into the cytoplasm, increase the cellular uptake of siRNA, prevent siRNA from being hydrolyzed, and promote its escape from lysosomes to achieve a better gene therapy effect [65]. C. Zhang et al. [60] constructed an artificial super neutrophil by wrapping GOx and CPO in ZIF-8 MOF nanoparticles. The crude neutrophil membrane was covered outside the nanoparticles, achieving effective inflammatory position targeting and hypochlorous acid generation. It has also been reported that the natural neutrophil membrane has a substantial inflammatory targeting influence, and GOx and CPO can undergo an enzymatic cascade reaction to catalyze the production of hypochlorite from glucose acid. The granulocyte membrane mixed with dual-enzyme system utilizes inflammatory targeting and hypochlorous acid-producing capabilities to target tumors and eliminate pathogens. In addition, the efficiency of hypochlorous acid generation using granulocytes is seven times higher than that using crude neutrophils. Therefore, it can be effectively used in anticancer and antimetastatic applications.

In summary, MOFs have several excellent properties and great potential in cancer therapy. In-depth research on antitumor MOFs with good biocompatibility, specific targeting, minor side effects, efficiency, safety, and economical treatment effects holds promise for the development of therapeutics against tumors.

## 5. Conclusion and Prospect

Despite significant breakthroughs in tumor treatment using biofunctionalized materials such as MOFs, the structural and performance advantages of MOFs have not been thoroughly investigated. There are limitations in the following aspects that need to be addressed. First, it is essential to optimize the design and synthesis of materials and develop medicine carriers with good biocompatibility. Second, the MOF drug carrier needs more prolonged blood circulation and is efficiently excreted by metabolism. Third, the potential toxic effects on organisms by the metal ions and organic ligands generated during the degradation of MOFs should be assessed. Fourth, the kinetic basis of drug release from MOFs as ordered porous materials is mainly based on host-guest interactions. Therefore, the release profile of this drug cannot be accurately controlled since the activation energy level required to overcome the host-guest interaction force is typically discrete and not continuously regulated. Fifth, the size of MOFs significantly affects the tumor penetration and retention capabilities of drugs, so a rational design is required for the size regulation of MOF carriers. Finally, the MOF drug system can be precisely designed to achieve controllable allosteric and functional functions in tumor treatment by comprehensively utilizing the pathological and physiological characteristics of the TME and the structural characteristics of the MOF materials, which could lead to a breakthrough in tumor treatment.

## Figures and Tables

**Table 1:** Summary of ROS-responsive MOFs/MOF-derivative drug carrier.

Compounds	Ref.
BSA/SAs-NMOF	[24]
MOF-199	[25]
MOF-QDs	[26]
PCN-822 (M) (M = Zr, Hf)	[27]
Sm-TCPP	[28]
Sm-TCPP-Pt/TPP	[28]
Bio-MOF-1&RCs	[30]
TPZ/Hf-TCPP/PEG	[31]
nMOF-Ti-TBP	[32]
Mn-ZrMOF nanocubes	[33]

**Table 2:** Summary of reduction-responsive nanodrug carriers.

Compounds	Ref.
(CuL-[AlOH]_2_)*_n_*	[34]
PS@MOF-199 NPs	[25]
OCZCF	[39]
UPFB/PCN-224 (Fe)	[40]
TUDMP	[41]
NS@TPZ (NST) NPs	[42]
Glud-MFo-c	[44]
NMIL-100	[46]
Sol/Mel@HKUST-1 NPs	[47]

**Table 3:** Summary of MOF drug loading.

MOF	Drug	Ref.
ZIF-8	Pentafluorouracil	[49]
MOF-In1	Pentafluorouracil	[50]
UiO-66	Polypyrrole nanoparticles	[51]
UiO-68 NMOFs	Doxorubicin hydrochloride	[52]
ZIF-90	Doxorubicin hydrochloride	[53]
ZIF-8	Doxorubicin hydrochloride	[54]
CBD/Mg-GA	Cannabidiol	[55]
nanoMOF	Gemcitabine-monophosphate	[56]
nanoMOF	Nitrosothiols	[57]
ZIF-8	Cas9	[58]
ZIF-8	DNA zyme	[59]
ZIF-8	GOx and CPO	[60]
ZIF-90	Cas9	[61]
